# Duplicated Renal System with H Shaped Ureter: An Extraordinary Anomaly

**DOI:** 10.1155/2016/4062515

**Published:** 2016-01-28

**Authors:** Fatih Akbulut, Metin Savun, Burak Ucpinar, Murat Sahan, Burak Arslan, Faruk Ozgor, Abdulmuttalip Simsek, Ahmet Yalcın Berberoglu, Murat Baykal, Murat Binbay

**Affiliations:** Haseki Training and Research Hospital, Department of Urology, 34096 Istanbul, Turkey

## Abstract

Duplex collecting systems are the most commonly encountered anomaly of the urinary system. Complete duplex system with an H shaped ureter is a very rare situation. There are only two reported H ureter cases in the literature. Herein, we aimed to present an H shaped ureter case, which was identified while performing ureterorenoscopy to a 48-year-old female patient due to a right distal ureteral stone.

## 1. Introduction

Duplex collecting systems are one of the most commonly encountered anomalies of upper urinary system and can be seen with anomalies like ectopic ureter, ureterocele, and vesicoureteral reflux but can also present as a normal functioning kidney with complete or partial duplication. Duplex collecting systems were identified in 0.8% of autopsy series and were more commonly seen in women (1.6/1). Presence of a unilateral duplex system is 6 times more common than bilateral duplex systems and anomaly is commonly identified on the right side [1]. Complete duplication is described as presence of two separate ureteral orifices opening into the bladder and partial duplication is described as fusion of two ureters before entering the bladder and opening into the bladder with a single ureteral orifice.

Fusion of two ureters at the midureter level with a bridge, complete duplex system with an H shaped ureter appearance is a very rare situation. There are only two reported H ureter cases in the literature [2, 3]. Herein, we aimed to present an H ureter case, which was identified while performing ureterorenoscopy to a 48-year-old female patient due to a right distal ureteral stone.

## 2. Case Presentation

**Figure 1:**
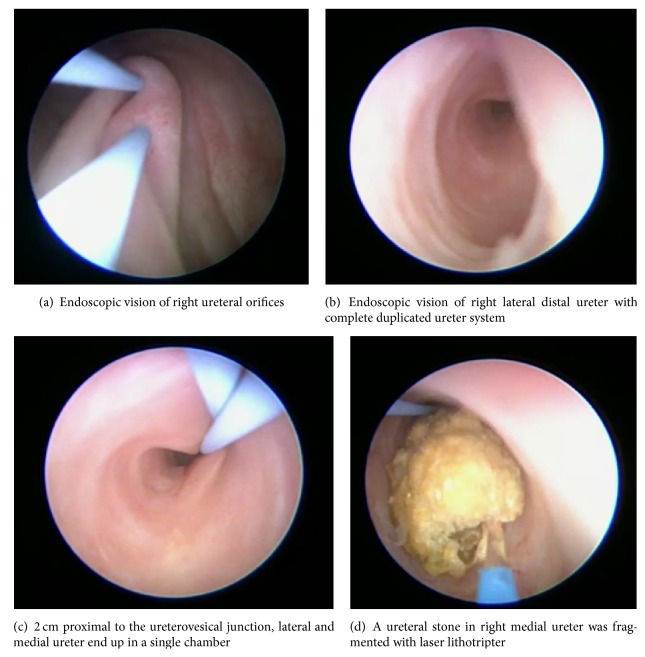
Complete duplicated system with H shaped ureter.

48-year-old female patient admitted to our outpatient clinic with intermittent right flank pain. She had a 30-year history of urolithiasis and had undergone multiple bilateral ureterorenoscopy operations and shock wave lithotripsy sessions. A right ureteral stone was suspected in kidney, ureter, and bladder urography and a noncontrast computed tomography revealed a 1 cm right distal ureteral stone and a millimetric calculi in middle pole of left kidney. Creatinine level of our patient was 0.8 mg/dL and sterile urine culture was obtained preoperatively. Ureterorenoscopy was planned for the patient. During the cystoscopy of the patient, two right ureteral orifices were identified. 0.038-inch guidewires (Boston Scientific, MA, USA) were introduced into both right ureteral orifices. The ureterorenoscope was inserted into the right lateral ureteral orifice via a guidewire. After advancement of the ureterorenoscope 2 cm proximal to the ureterovesical junction, lateral and medial ureter end up in a single chamber. After this, they were separated again and continued proximally as two separated ureters. Laterally introduced guidewire was crossing to the medial ureter and medially introduced guidewire was crossing to the lateral ureter. Retrograde pyelogram was performed and H shaped ureter was visualized. 1 cm-sized calculus was identified in the right medial ureter. (Figure 1) Stone was fragmented with laser and fragments were collected with a hydrophilic basket. Operation was terminated by placing a double J stent into the right medial ureter. Double J stent was removed 3 weeks later.

## 3. Discussion

Duplex collecting systems are the most commonly encountered anomaly of the urinary system. Anomalies like ureterocele and ectopic ureter, which affects the upper system, and anomalies like ureteropelvic junction obstruction and vesicoureteral reflux, which affects the lower system, can be encountered in patients with duplex systems [4]. Therefore, early diagnosis of duplex systems is highly important.

When the ureteric bud prematurely divides before penetrating the metanephric blastema, this results in an incomplete duplex with ureters that meet before the bladder or a bifid renal pelvis. If more than one ureteric bud develops and migrates to the metanephric blastema, a duplex kidney with two separate ureters and orifices occurs. The ureteric bud at the cranial side connects to the upper pole. However, as the ureteric bud incorporates into the bladder, the upper pole ureteric bud rotates and migrates more caudally than the lower pole ureteric bud. This results in the upper pole ureter having an opening that is more caudal than the lower pole ureter (Weigert Meyer Rule) [5]. In our case, two ureters progressed 2 cm separately in distally; then they end up in a single chamber. After this, they were bifurcated into two ureters in the proximal part. In the first case of H shaped ureter, Jaysekara et al. speculated that the two ureteric buds may have fused close to their origin on the Wolffian duct and then bifurcated before reaching the metanephric blastema, to account for this anomaly [2].

Erosion of impacted stones through the wall of the ureter has been reported previously [6]. Erosion of the wall of two ureters due to impacted stones and multiple ureterorenoscopy surgery could be the explanation of the forming of a single chamber. In our case, the patient had history of multiple ureterorenoscopic surgeries in her life.

We have encountered two H ureter cases in the literature [2, 3]. They were both identified during right ureterorenoscopy due to a right ureteral stone and ureteral chamber and were both located close to the ureterovesical junction.

Visualizing the ureter directly during ureterorenoscopy can identify ureteral anomalies but imaging methods are also beneficial for identifying this sort of anomalies. CT scan with urographic phase can be a valuable imaging method but we did not have any suspicion of an anomaly in our case; thereby, we have obtained a CT scan without contrast. However, retrograde pyelogram should be performed during surgery and lack of our retrograde pyelogram images and a CT scan with urographic phase can be listed as limitations of our case presentation.

Patients with complete duplex systems may have multiple concomitant urinary tract anomalies and H ureter is one of the rarest ones among those anomalies. Urologists should keep this anomaly in mind when they encounter a patient with duplex system. To the best of our knowledge, this is the 3rd H ureter case in the literature.
